# Different preoperative fluids do not affect the hemodynamic status but gastric volume: results of a randomized crossover pilot study

**DOI:** 10.1186/s12871-022-01697-3

**Published:** 2022-05-24

**Authors:** Shuhua Zhao, Qiong Ling, Fengping Liang, Zhongmei Lin, Yingqing Deng, Shaonong Huang, Qianqian Zhu

**Affiliations:** 1 Department of Anesthesiology, The Seventh Affiliated Hospital of Sun Yat-Sen University, Shenzhen City, Guangdong Province 518107 People’s Republic of China; 2 Guangzhou Medical University, No.1 Xinzao Road, Panyu District, Guangzhou City, 511436 People’s Republic of China; 3 Department of Anesthesiology, The Second Affiliated Hospital of Guangzhou University of Chinese Medicine, Guangzhou City, People’s Republic of China; 4 Department of Medical Ultrasound, The Seventh Affiliated Hospital of Sun Yat-sen University, Shenzhen City, People’s Republic of China

**Keywords:** IVC, IVCCI, Gastric volume

## Abstract

**Background and objective:**

Inferior vena cava (IVC) examination has been reported as a noninvasive method for evaluating the hemodynamic state. We conducted this crossover pilot study to investigate the effects of the administration of water and high-carbohydrate-containing fluids on the hemodynamic status of volunteers through collapsibility index of IVC (IVCCI) measurement.

**Methods:**

Twenty volunteers were randomly assigned to a water or high-carbohydrate group according to computer-generated random numbers in a 1:1 ratio. In the water group, volunteers received water (5 mL/kg), and in the high-carbohydrate group, patients received carbohydrate drinks (5 mL/kg). Respiratory variations in the IVC diameter, gastric volume, and blood pressure and heart rates in erect and supine positions were measured at admission (T1), 1 h (T2), 2 h (T3), 3 h (T4), and 4 h (T5).

**Results:**

When considering participants with an IVCCI of more than 42%, there were no significant differences between the water and carbohydrate drink groups at each time point (all *p* > 0.05). At T2, more participants had an empty stomach in water group than in carbohydrate drink group (*p* < 0.001). At T3, 30% of the participants could not empty their stomachs in carbohydrate drink group. However, with regard to the number of volunteers with empty stomach at T3, there was no significant difference between water and carbohydrate drink group. Repeated measures data analysis demonstrated that IVCCI showed no significant differences over time (*p* = 0.063 for T1-T5). There were no differences between water and carbohydrate drinks (*p* = 0.867).

**Conclusion:**

Our results suggested that neither water nor carbohydrate drinking affected the hemodynamic status through IVCCI measurement over time, up to 4 h after drinking. Furthermore, carbohydrate drinking might delay gastric emptying at 1 h, but not 2 h after drinking, in comparison with water.

**Supplementary Information:**

The online version contains supplementary material available at 10.1186/s12871-022-01697-3.

## Introduction

Prolonged fasting might result in unstable hemodynamics, which could be attributed to low blood volume with conflicts [1–3]. Inferior vena cava (IVC) examination has been reported as a noninvasive method to evaluate the hemodynamic state and predict the response to fluid therapy [4–6]. The use of collapsibility index of IVC (IVCCI) to assess hemodynamic state was more helpful in spontaneously breathing patients than those who were mechanically ventilated in previous study [7].

In comparison with pure water, a clear fluid containing a relatively high concentration of complex carbohydrates 2 h before surgery could provide a more comfortable feeling [8]. Protein balance and muscle strength could also be well maintained in patients who received carbohydrate-containing fluids preoperatively [9, 10]. Previous study demonstrated that oral intake of fluid type influenced acute hydration and muscle performance recovery [11]. Weather different oral intake of fluid type showed different blood volume keep ability remained unclear. To the best of our knowledge, no study has compared the hemodynamic states between population fasting after different fluids (water and high-carbohydrate-containing fluids) through IVC-related examinations. We conducted the present pilot study to investigate the effects of the administration of water and high-carbohydrate-containing fluids on the hemodynamic status of volunteers.

## Materials and methods

This randomized pilot crossover study was performed in accordance with the Declaration of Helsinki. This study was approved by the Institutional Review Board of the Seventh Affiliated Hospital of Sun Yat-sen University (approval number: KY-2020-010-01) and registered with the Chinese Clinical Trial Registry at www.chictr.org (registration date:26/05/2021, registration number: ChiCTR2100046656). This manuscript adhered to the applicable CONSORT 2010 checklist.

Volunteers were recruited from June 1st, 2021, and the procedure of data collected were performed from June 12 to June 28, 2021. Twenty volunteers participated in this study. The inclusion criteria were: 1) age 18–60 years, 2) no history of chronic diseases including gastrointestinal dysfunction, and 3) obtaining informed written consent. Participants who met the following criteria were excluded: 1) BMI > 28 km/m^2^ or BMI < 18 km/m^2^, 2) pregnant women or who breastfed, and 3) taking medication that could affect esophageal or gastric function. All volunteers were offered financial compensation for their participation.

Subjects were randomly assigned to the water or high carbohydrate groups by SZ according to computer-generated random numbers in a 1:1 ratio. The numbers were sealed in envelope until the participants received intervention.

Study participants fasted for 6–8 h of solid food, and were free to water 2 h before the study. In the water group, volunteers received water (5 mL/kg), and in the high-carbohydrate group, patients received carbohydrate drinks (5 mL/kg containing 12.5% carbohydrates). All the drinks were prepared in bottles with the same appearance and were distributed by the same trial assistant who was not involved in the follow-up or assessment. Blood pressure in the erect and supine positions, respiratory variations in the inferior vena cava (IVC) diameter, and gastric volume were measured at admission (T1), 1 h (T2), 2 h (T3), 3 h (T4), and 4 h (T5) after drinking. Thirst (0, no thirst; 10, unbearable thirst), and hunger (0, no hunger; 10, unbearable hunger) were assessed using a visual analog score based on that of a previous study at each time point [8]. The timeline of the procedure is shown in Supplementary document 1.

Inferior vena cava (IVC) diameters were recorded at the end of expiration (IVCmax) and inspiration (IVCmin) using a 1–5 MHz transducer (C5–1) and a Philips Medical system (Philips Medical system, Bothell, Washington, USA). The primary outcome was the collapsibility index of IVC (IVCCI) of the two groups. The IVCCI was calculated as follows: IVCCI = (IVCmax-IVCmin)/IVCmax*100%. In reference to a previous study, IVCCI > 42% was considered to be responsible for the fluid response in spontaneously breathing patients [12].

The cross-sectional areas (CSA) of the antrum, body, and fundus were determined using a curvilinear array 1–5 MHz transducer (X5–1) and a Philips Medical system (Philips Medical system, Bothell, Washington, USA), referring to a previous study [13]. The gastric volume was calculated according to the following model, referring to a previous study: Volume = 27.0 + 14.6 * Right − latCSA − 1.28 * age [14]. An empty stomach was defined as a resident gastric volume of less than 0.8 ml/kg, which was the cutoff volume in cases of regurgitation [15].

Ultrasonic-related parameters were measured independently. If the parameter difference were more than 20%, a third investigator would measure it again. The final data was the mean values of two measures. If there were three measures, the outlier was abandoned.

### Sample size

There was no study involved in IVC related index to compare water and high-carbohydrate-containing fluids on the hemodynamic status. Therefore, in order to explore the use of IVC related index in patients in the future study, a pilot study was designed first. According to results of we observed, about 60% patients has a IVCCI more than 42%. Therefore, we assumed 60% participants has a IVCCI more than 42% 4 h after taking water, 30% 4 h after taking carbohydrate drinks. Therefore, 20 participants were required in each group for a power of 80% and a two-tailed *p*-value of 0.05 was considered as statistically significant in a crossover study. For it was pilot study, we also refer to previous pilot study [16, 17], and recruited 20 participants in present study.

### Statistical analysis

Qualitative data were expressed as percentage/composition ratios, and Pearson’s chi-squared test or Fisher’s exact probabilities were used to compare differences. Quantitative data were expressed as the mean ± standard deviation (SD) or range, which would be indicated. The general linear model (GLM) was also used to assess the differences between water and carbohydrate drinks at each time point. Repeated measures data analysis was also used to compare the variation of IVCCI before and after drinking at different time points between the two groups. The response variable was the difference in the IVCCI variation with two factors: group (intervention) and time (before and after drinking). The interaction (group × time) was evaluated if the effects were different between water and carbohydrate drinks over time. Differences with *p* < 0.05 (two-tailed) were considered statistically significant. Statistical analyses were performed using SPSS version 19.0 (IBM Corporation, Armonk, NY, USA).

### Patient and public involvement

No patients or public members were involved in the development of the research question or recruitment or outcome measures nor the design of the study. There are no plans to disseminate the results of the research to study participants.

## Results

Nine women and 11 men, with a mean ± SD age of 24.55 ± 3.41 years (range 21–31 years) and a mean ± SD body mass index (BMI) of 21.08 ± 1.94 kg/m^2^ (range, 18.33–25.54 kg/m^2^), participated in the present study (Table 1). No participant was lost to follow up. The procedure of inclusion is shown in flow diagram (Supplementary document 2).

**Table 1:** The characteristics of included volunteers

Patient	Gender	Age (y)	Weight (kg)	Height (m)	BMI (kg/m^2^)
1	M	21	70	1.7	24.22
2	F	24	47	1.57	19.07
3	M	22	77	1.87	22.02
4	M	23	70	1.73	23.39
5	M	23	55.5	1.74	18.33
6	M	28	61.6	1.77	19.66
7	M	24	62	1.77	19.79
8	F	22	61	1.63	22.96
9	M	22	72.5	1.83	21.65
10	F	21	57	1.58	22.83
11	F	29	49	1.6	19.14
12	F	22	55.5	1.68	19.66
13	M	21	62	1.7	21.45
14	F	22	53	1.6	20.70
15	F	31	50	1.55	20.81
16	F	28	53	1.63	19.95
17	M	28	61	1.7	21.11
18	M	30	80	1.77	25.54
19	F	28	46	1.57	18.66
20	M	22	62	1.73	20.72

*BMI* Body mass index

### IVCCI

When considering participants with an IVCCI of more than 42%, there were no significant differences between the water and carbohydrate drink groups at each time point (all *p* > 0.05, Table 2). Considering the number of IVCCI of more than 42% change over time, there were no significant changes in water (*p* = 0.543 for T1-T5, *p* = 0.913 for T2-T5, Table 2) or in the carbohydrate group (*p* = 0.330 for T1-T5, *p* = 0.414 for T2-T5, Table 2).

**Table 2:** The comparison between water and carbohydrate group

	Time point	Water (20)	Carbohydrate (20)	p
IVCCI> 42%	T1	8 (40%)	6 (30%)	0.507
	T2	6 (30%)	6 (30%)	1.000
	T3	5 (25%)	5 (25%)	1.000
	T4	6 (30%)	4 (20%)	0.465
	T5	6 (30%)	4 (20%)	0.465
	p linear (T1 to T5)	0.543	0.330	
	p linear (T2 to T5)	0.913	0.414	
GV < 0.8 ml/kg	T1	18 (90%)	16 (80%)	0.658
	T2	16 (80%)	4 (20%)	< 0.001
	T3	18 (90%)	14 (70%)	0.236
	T4	20 (100%)	19 (95%)	1.000
	T5	20 (100%)	19 (95%)	1.000
	p linear (T1 to T5)	0.038	0.001	
	p linear (T2 to T5)	0.008	< 0.001	

*IVCCI* Collapsibility index of Inferior vena cava

*GV* Gastric volume

**Fig. 1:**
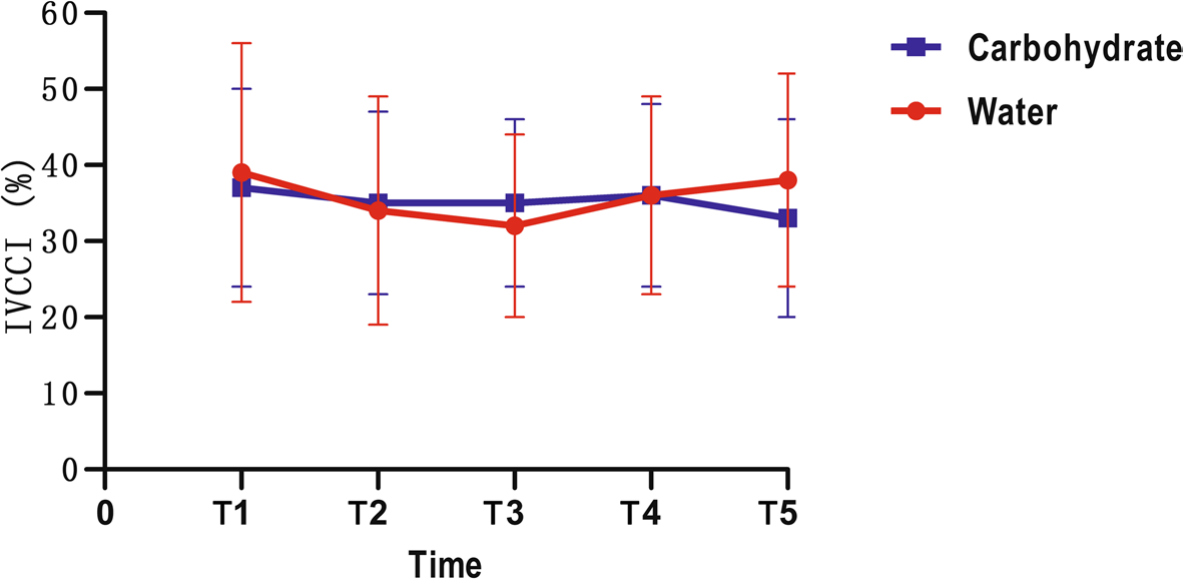
The IVCCI of different time points. IVCCI: collapsibility index values of Inferior vena cava

In GLM univariate analyses, the IVCCI showed no significant differences at each time point between the water and carbohydrate drink groups either (all *p* > 0.05, Fig. 1). Repeated measures data analysis demonstrated that IVCCI showed no significant differences over time (*p* = 0.063 for T1-T5, Fig. 1, Table 3). There were no differences between the water and carbohydrate drinks (*p* = 0.867, Table 3).

**Table 3:** Comparison the variation of parameters before and after drinking at different time points between the two groups

	Time	Water	Carbohydrate
IVCCI	T1	0.39 ± 0.17	0.37 ± 0.13
	T2	0.34 ± 0.15	0.35 ± 0.12
	T3	0.32 ± 0.12	0.35 ± 0.11
	T4	0.36 ± 0.13	0.36 ± 0.12
	T5	0.38 ± 0.14	0.33 ± 0.13
	p time (T1 to T5) = 0.063		p between groups = 0.867
GV (ml)	T1	18.93 ± 30.37	27.89 ± 28.37
	T2	27.98 ± 34.63	110.65 ± 73.90
	T3	23.11 ± 26.56	38.74 ± 41.11
	T4	27.97 ± 19.87	20.29 ± 20.69
	T5	24.27 ± 17.86	34.93 ± 33.49
	p time (T1 to T5) = 0.285		p between groups = 0.005
Supine SBP (mmHg)	T1	118.50 ± 12.21	112.60 ± 11.24
	T2	111.90 ± 11.17	114.50 ± 10.02
	T3	113.45 ± 11.69	114.30 ± 13.27
	T4	115.25 ± 10.64	108.80 ± 8.74
	T5	114.75 ± 9.95	113.95 ± 12.56
	p time (T1 to T5) =0.955		p between groups = 0.551
Supine DBP (mmHg)	T1	70.65 ± 9.30	71.85 ± 8.05
	T2	68.65 ± 8.67	69.30 ± 7.59
	T3	69.60 ± 8.46	70.70 ± 6.99
	T4	73.10 ± 9.77	68.65 ± 7.07
	T5	69.65 ± 8.99	70.10 ± 8.40
	p time (T1 to T5) =0.809		p between groups = 0.926
Supine HR (bpm)	T1	72.05 ± 13.69	70.75 ± 10.19
	T2	66.40 ± 10.94	71.90 ± 8.93
	T3	64.85 ± 9.72	70.20 ± 7.98
	T4	66.15 ± 10.09	64.40 ± 7.24
	T5	65.75 ± 9.65	66.75 ± 8.85
	p time (T1 to T5) =0.309		p between groups = 0.513
Orthostatic SBP (mmHg)	T1	118.45 ± 12.55	117.20 ± 10.57
	T2	114.75 ± 11.62	117.80 ± 12.32
	T3	116.00 ± 10.48	115.05 ± 12.60
	T4	116.65 ± 10.00	112.30 ± 8.85
	T5	117.25 ± 12.78	117.25 ± 14.28
	p time (T1 to T5) =0.381		p between groups = 0.827
Orthostatic DBP (mmHg)	T1	77.75 ± 9.11	78.10 ± 6.40
	T2	76.45 ± 8.17	76.60 ± 7.47
	T3	77.30 ± 7.58	76.70 ± 6.52
	T4	77.65 ± 8.00	75.00 ± 6.91
	T5	77.60 ± 6.40	75.10 ± 7.29
	p time (T1 to T5) =0.408		p between groups = 0.602
Orthostatic HR (bpm)	T1	78.20 ± 13.82	79.35 ± 13.85
	T2	74.75 ± 12.83	79.30 ± 10.44
	T3	72.70 ± 10.29	76.70 ± 11.29
	T4	74.60 ± 9.60	71.65 ± 9.99
	T5	72.70 ± 11.68	71.75 ± 10.06
	p time (T1 to T5) =0.191		p between groups = 0.700
Thirst Score	T1	1.60 ± 0.60	1.45 ± 0.60
	T2	1.35 ± 0.49	1.30 ± 0.47
	T3	1.40 ± 0.50	1.50 ± 0.61
	T4	1.45 ± 0.51	1.50 ± 0.51
	T5	1.60 ± 0.50	1.70 ± 0.57
	p time (T1 to T5) =0.489		p between groups = 0.938
Hunger Score	T1	1.50 ± 0.51	1.55 ± 0.51
	T2	1.75 ± 0.64	1.30 ± 0.47
	T3	1.70 ± 0.57	1.40 ± 0.50
	T4	1.80 ± 0.52	1.65 ± 0.59
	T5	1.90 ± 0.55	2.00 ± 0.65
	p time (T1 to T5) =0.087		p between groups = 0.221

*IVCCI* Collapsibility index of Inferior vena cava

*GV* Gastric volume

*SBP* Systolic blood pressure

*DBP* Diastolic blood pressure

*HR* Heart rate

### Gastric volume

**Fig. 2:**
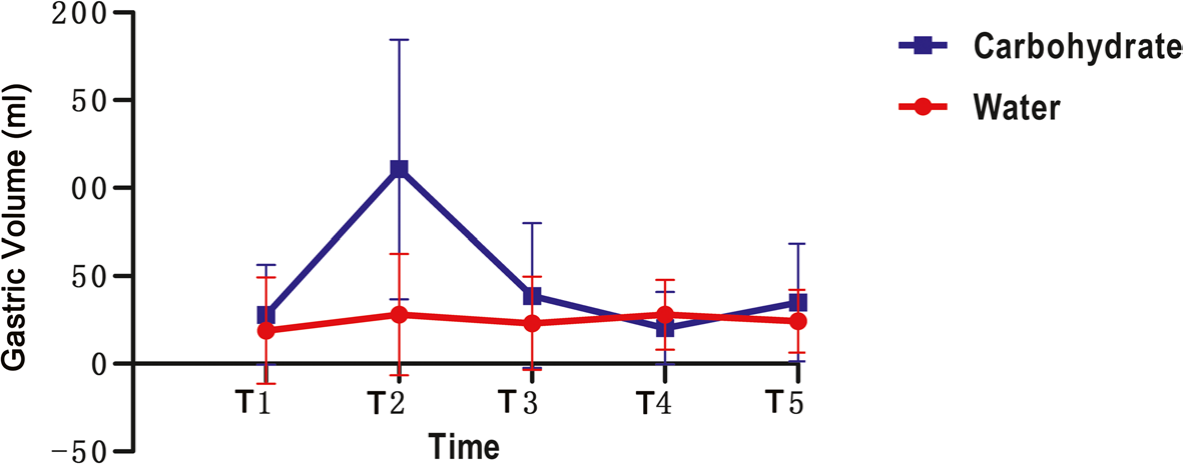
The gastric volume of different time points

The gastric volume was not significantly different between the volunteers in the water and carbohydrate drink groups at T1 (*p* = 0.237, Fig. 2). One hour after drinking (T2), more participants had an empty stomach (gastric volume less than 0.8 ml/kg) in the water group than in the carbohydrate drink group (*p* < 0.001, Table 2). Two hours after drinking, 30% of the participants could not empty their stomachs in the carbohydrate drink group (Table 2). However, with regard to the number of volunteers with empty stomach at T3, there was no significant difference between water and carbohydrate drink group (*p* = 0.236, Table 2).

The repeated measures data analysis showed that the gastric volume was statistical different between the volunteers after the water and carbohydrate drinking (*p* = 0.005, Table 3).

### Blood pressure, thirsty, and hunger score

**Fig. 3:**
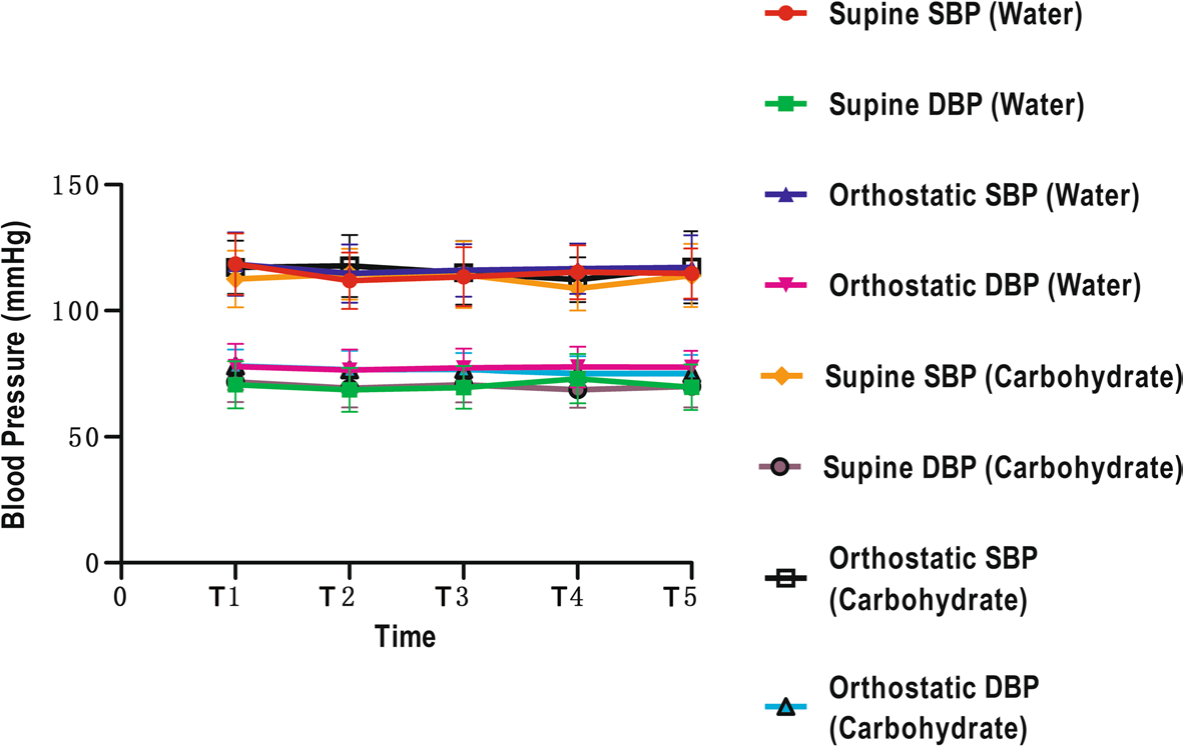
The blood pressure of different time points. SBP: systolic blood pressure; DBP: diastolic blood pressure

**Fig. 4:**
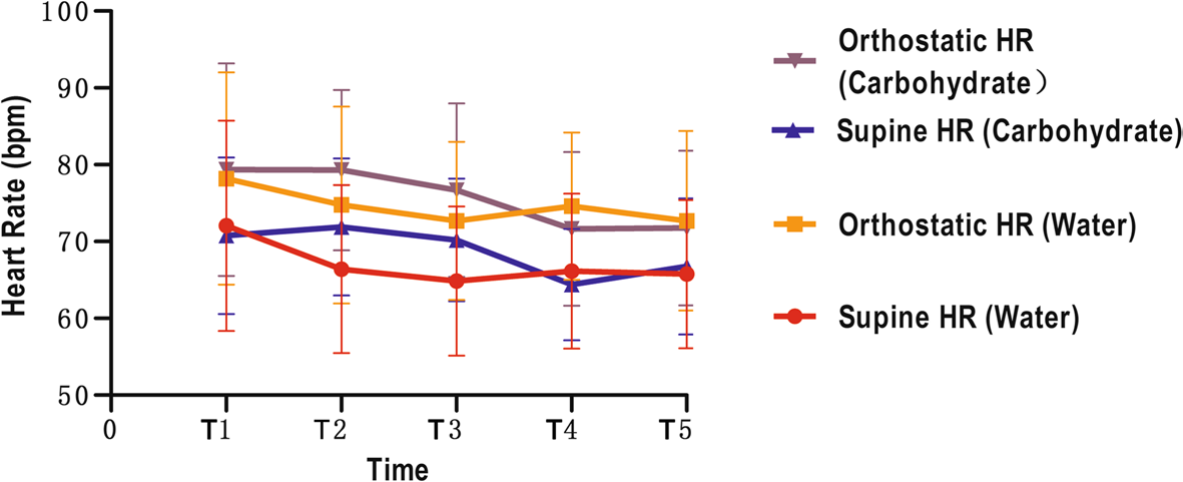
The heart rate of different time points. HR: Heart rate

**Fig. 5:**
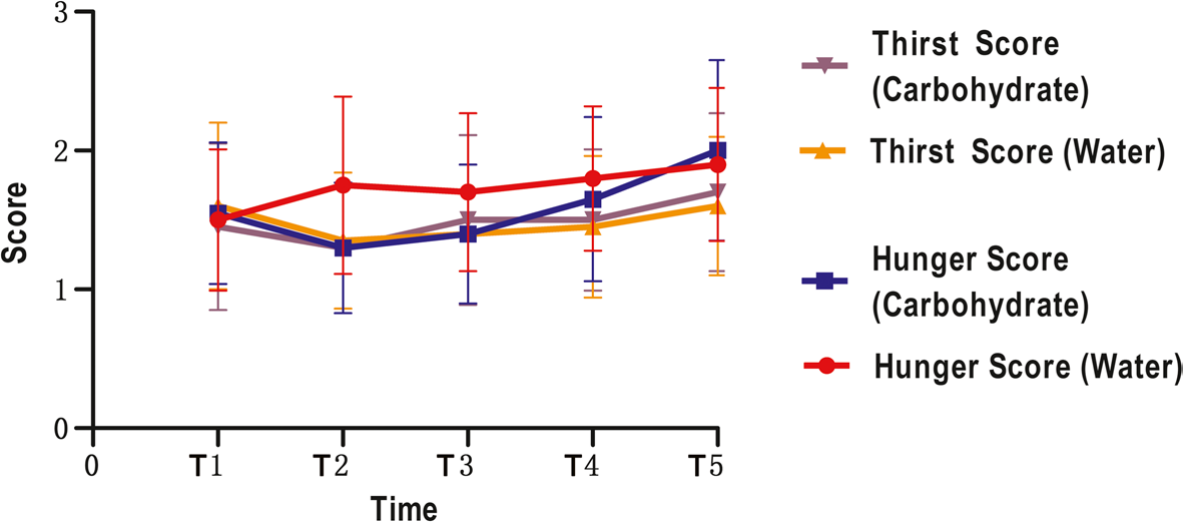
The thirsty and hunger score of different time points

There were no significant changes over time, or between the water and carbohydrate drinks when considering blood pressure (both *p* > 0.05 for T1-T5, Fig. 3, Table 3), heart rates (both p > 0.05 for T1-T5, Fig. 4, Table 3), and thirst and hunger scores (both p > 0.05 for T1-T5 and T2-T5, Fig. 5, Table 3). The results of GLM univariate analyses for each time point of were also provided in Table 3.

## Discussion

The results of the present study demonstrated that 5 ml/kg of water or carbohydrate drink did not affect the IVCCI for up to 4 h after drinking through IVCCI measurement. Moreover, blood pressure in the erect and supine positions was not affected by water or the carbohydrate drinks. Furthermore, the stomach could empty water faster than carbohydrates.

Preoperative fasting is aimed at avoiding pulmonary aspiration, but a long period of fasting was assumed to be related to hypovolemia and hypotension during anesthesia induction period [18, 19]. In contrast, other researchers indicated that preoperative fasting does not induce significant hypovolemia by passive leg rising in patients with ASA status I–III, according to the transthoracic echocardiography (TTE) criteria [3]. Hypotension during anesthesia induction was not related to preoperative fasting in healthy patients [20]. Even 12 hours of fasting before elective surgery did not result in hypovolemia [21]. The present study suggested that no IVCCI changes occurred over time after 5 ml/kg of clear fluids, including water or carbohydrate drinking, for up to 4 h in the healthy volunteers. Considering those with an IVCCI greater than 42%, who might be fluids responders, there were no significant changes with time in either the water or carbohydrate group. Arterial pressure and heart rate were not significantly affected. The results indicated that 4-h fasting after taking clear fluids might not affect the blood volume as assessed by IVCCI. It was hypothesized in a previous study, hypotension during anesthesia induction might be attributed to the effect of anesthesia on vascular tone rather than an absolute blood volume deficit [20]. However, future studies are needed to confirmed the association between fasting and hypovolemia or hypotension during anesthesia.

Current guidelines have recommended the free to intake of clear fluids until 2 h before anesthesia [22, 23]. In comparison with water, preoperative oral carbohydrate was indicated to have a suppressive effect on insulin resistance and balance whole-body protein, and has been recommended for up to 2–3 h before anesthesia [9, 24]. However, with regard to postoperative hospital stay and well-being, carbohydrate drinking before elective surgery was not superior to water [25–27]. Of note, no conclusion regarding the impact of preoperative carbohydrate drinks on gastric volume was reached [27]. Although a study suggested that carbohydrate drinks did not alter the amount of gastric contents in comparison with fasting [28, 29], the comparison of digestion kinetics between carbohydrate drinks and water, especially within 2 h, has not been investigated. The results of the present study demonstrated that gastric volume was significantly higher 1 h after carbohydrate drinking than water. At 2 h after drinking, the resident gastric volume did not differ between the carbohydrate drinking and water groups. Of note, most patients could empty water, but not carbohydrate drinks within 1 h.

However, this study had several limitations. First, the GSA and IVCCI by ultrasonography do not directly measure the gastric volume and blood volume. However, as a non-invasive tool, ultrasonography is widely used. Ultrasonography has been used as a fast and effective non-invasive tool for assessing blood volume in critically ill patients [30, 31]. However, results from critically patients demonstrated whether the IVC related index were reliable in reflect blood volume in spontaneous populations remains inconclusive [32, 33]. In spontaneously breathing individuals, an IVCCI of more than 40% or 42% in different studies could predict fluid responsiveness with conflict results. One study demonstrated that IVCCI more than 42% might predict an increase in cardiac output but not fluid responsiveness after fluid infusion in spontaneously breathing individuals with suspected hypovolemia [12]. In contrast, there were also studies suggested that an IVCCI of more than 40% were associated with fluid responsiveness, while an IVCCI of less than 40% or 42 did not rule out fluid needs [32, 34]. Therefore, the reliability of IVCCI use in predict fluid responses and the cut-off value of IVCCI to yield both high sensitivity and specificity were required to be confirmed in future studies. Furthermore, because of the potential ability in reflect blood volume and non-invasive peculiarity, the studies of IVCCI use in hemodynamic status measurement should be encouraged in future. With regard to the GSA measured by ultrasonography, it was not the direct gastric volume. However, the GSA in the right lateral decubitus position could also reflect gastric volume correctly [13, 14, 35]. Second, it was only a pilot study involving a small number of young volunteers. The results could not be generalized to aged individuals and need to be confirmed in further studies. The results could not be extrapolated to the patients undergoing general anesthesia neither. Third, the clear fluids in the present study were 5 ml/kg, and a fasting time was 4 h after drinking. The IVCCI changes could not be extrapolated to larger volumes of drinking or longer fasting duration.

In summary, our results from this crossover study including 20 volunteers suggested that neither water nor carbohydrate drinking affected the hemodynamic status through the IVCCI measurement over time for up to 4 h after drinking. Furthermore, carbohydrate drinking might delay gastric emptying at 1 h, but not 2 h after drinking, in comparison with water. Most patients could empty water, but not carbohydrate drinking, within 1 h. It was indicated that in comparison with water, carbohydrate drinking showed no superiority in maintaining blood volume or gastric emptying for up to 4 h after drinking by the volunteers. However, the results need to be confirmed by generalized studies involving patients of different ages and ASA statuses in future studies.

## Supplementary Information

**Additional file 1.**

**Additional file 2.**

## Data Availability

The data used to support the findings of this study are included within the article.
