# Supplementary figures and images for: Different preoperative fluids do not affect the hemodynamic status but gastric volume: results of a randomized crossover pilot study
Source: BMC Anesthesiol. 2022 May 24;22:158. doi: 10.1186/s12871-022-01697-3 (PMC9128243; doi:10.1186/s12871-022-01697-3)

# The timeline of the procedure

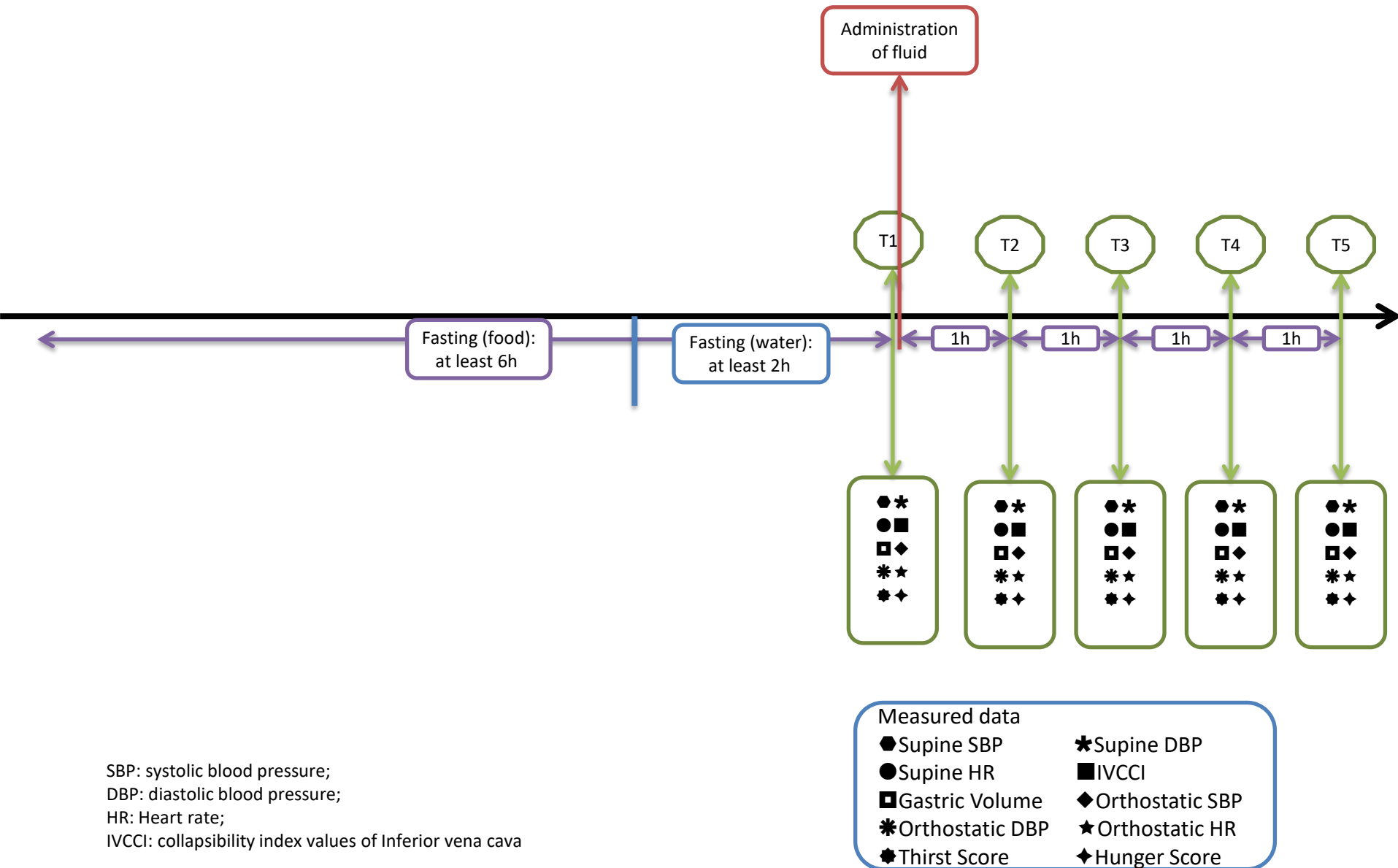

Supplement: Supplementary file 1 — Additional file 1. [file 12871_2022_1697_MOESM1_ESM.pdf]
