# Supplementary material for: Different preoperative fluids do not affect the hemodynamic status but gastric volume: results of a randomized crossover pilot study
Source: BMC Anesthesiol. 2022 May 24;22:158. doi: 10.1186/s12871-022-01697-3 (PMC9128243; doi:10.1186/s12871-022-01697-3)
Supplement: Supplementary file 2 — Additional file 2. [file 12871_2022_1697_MOESM2_ESM.doc]

**CONSORT 2010 Flow Diagram**

**Allocation**

**Analysis**

**Follow-Up**

**Enrollment**

**Crossover**

Assessed for eligibility (n=20)

Excluded (n=0)

♦  Not meeting inclusion criteria (n=0)

♦  Declined to participate (n=0)

♦  Other reasons (n=0)

Analysed (data of 20 volunteers drinking water)
♦ Excluded from analysis (n=0)

Lost to follow-up (n=0)

Discontinued intervention (n=0)

Allocated to water (n=10)

♦ Received allocated intervention (n=10)

♦ Did not receive allocated intervention (n=0)

Lost to follow-up (n=0)

Discontinued intervention (n=0)

Allocated to carbohydrate (n=10)

♦ Received allocated intervention (n=10)

♦ Did not receive allocated intervention (n=0)

Analysed (data of 20 volunteers drinking carbohydrate)
♦ Excluded from analysis (n=0)

Randomized (n= 20)
